# 20-Hydroxyecdysone ameliorates metabolic and cardiovascular dysfunction in high-fat-high-fructose-fed ovariectomized rats

**DOI:** 10.1186/s12906-020-02936-1

**Published:** 2020-05-06

**Authors:** Jariya Buniam, Natsasi Chukijrungroat, Yupaporn Rattanavichit, Juthamard Surapongchai, Jittima Weerachayaphorn, Tepmanas Bupha-Intr, Vitoon Saengsirisuwan

**Affiliations:** 1 Department of Physiology, Faculty of Science, Mahidol University, Bangkok, 10400 Thailand; 2 Faculty of Physical Therapy, Huachiew Chalermprakiet University, Samut Prakan, 10540 Thailand; 3 Division of Physical Therapy, Faculty of Physical Therapy, Srinakharinwirot University, Nakhon Nayok, 26120 Thailand; 4 Faculty of Physical Therapy, Mahidol University, Nakhonpathom, 73170 Thailand

**Keywords:** 20-hydroxyecdysone, Ovariectomy, High-fat high-fructose diet, Glucose metabolism, Cardiometabolic syndrome

## Abstract

**Background:**

Ecdysteroids are polyhydroxylated steroids present in invertebrates and plants. 20-Hydroxyecdysone (20E) is the most common and the main biologically active compound of ecdysteroids. Previous studies have demonstrated anabolic and metabolic effects of 20E in mammals. However, it is unknown whether 20E has a positive effect on all aspects of cardiometabolic syndrome. The aims of this study were to investigate the favorable effect and possible underlying mechanisms of 20E in a rat model of cardiometabolic syndrome (CMS) induced by a high-calorie diet combined with female sex hormone deprivation.

**Methods:**

20E (5 mg/kg, 10 mg/kg, or 20 mg/kg) or pioglitazone (PIO) (10 mg/kg) was intragastrically administered to sham-operated Sprague-Dawley female rats and ovariectomized rats fed a high-fat-high-fructose diet (OHFFD) for 8 weeks. The phenotypic characteristics of CMS, including central adiposity, blood pressure, serum lipid profile, glucose tolerance, insulin action on skeletal muscle glucose transport activity and hepatic protein expression, were determined.

**Results:**

Some CMS characteristics were improved by 20E treatment. Rats treated with 20E had lower body weight, abdominal fat accumulation than rats treated with vehicle control without changes in total caloric intake and fat-free mass. OHFFD rats exhibited high blood pressure, but 20E-treated rats maintained normal blood pressure with a lower level of low-density lipoprotein (LDL)-cholesterol. Although 20E showed no positive effect on inducing insulin-mediated glucose transport in the skeletal muscle of OHFFD rats, 20E improved whole body glucose homeostasis. Analysis of protein expression in livers from 20E-treated rats revealed significantly increased expression of pAkt Ser^473^, pFOXO1 Ser^256^, pAMPKα Thr^172^, and FGF21.

**Conclusion:**

20E treatment can alleviate cardiometabolic disorder caused by a high-fat-high-fructose diet and female sex hormone deprivation. In particular, 20E helps improve whole body insulin sensitivity in OHFFD rats, and the mechanisms that underlie this favorable effect are potentially mediated by the activation of AMPK and FGF21. The present study indicates that 20E could be an alternative therapeutic option for the prevention and alleviation of cardiometabolic syndrome.

## Background

Cardiometabolic syndrome (CMS) is a clustering of multifaceted conditions characterized by several cardiovascular and metabolic risk factors, including dyslipidemia, hypertension, central obesity, compensatory hyperinsulinemia, and glucose intolerance [1, 2]. One of the major causes of CMS is excessive caloric intake or alterations in diet composition. Fat intake appears to be an important determinant of obesity. In addition, an increase in fructose consumption, which is largely attributable to sweetened beverages, can lead to CMS [3–6]. Typically, the prevalence of CMS is higher in females after menopause [7, 8]. However, currently, there are no specific medications that can prevent cardiometabolic dysfunction.

The pharmaceutical approach to CMS needs to be considered from various perspectives. Anti-diabetes agents are used as a broad base approach to induce whole-body insulin sensitivity and reduce the CMS phenotype. One of the most common clinical drugs prescribed to CMS patients is pioglitazone (PIO). Unfortunately, prolonged use of PIO may induce unfavorable effects, including edema, pulmonary edema, hepatic steatosis, and congestive heart failure [9, 10]. Thus, CMS medication discovery and development should preferably be shifted toward natural sources without side effects [9]. Notably, 20E is one of the candidates that has been studied [11–14]. Ecdysteroids are known as invertebrate steroid hormones that are involved in all stages of development, including newly laid eggs, embryogenic development, metamorphosis, reproduction and diapause [15]. Additionally, ecdysteroids can be found in many plant species around the world (phytoecdysteroids) [14, 15]. The major biologically active ecdysteroid found in invertebrates and plants is 20-hydroxyecdysone (20E) [12]. 20E is normally available in large amounts in certain plants, some of which are used for human food, such as spinach and quinoa [13]. A number of studies have revealed many potential beneficial health effects of 20E including the wound-healing, immunoprotective and anti-osteoporosis effects [12–15].

The early finding of the underlying mechanism was projected to be the anabolic effect of 20E [11]. For example, 20E was shown to increase protein synthesis in skeletal muscle [16] and increase physical endurance [17] in rodents. Furthermore, it has been reported that 20E is involved in metabolic effects in cell-based and animal-based studies [14, 15, 18]. The hypoglycemic [19, 20] and anti-obesity effects [21, 22] of 20E have been demonstrated in HepG2 cells and obese rodents, respectively. The findings on the metabolic effects of 20E revealed that 20E could prevent hyperglycemia in insulin-resistant rats by decreasing hepatic glucose consumption [19, 23]. More recent studies also showed that daily oral administration of 20E for 13 weeks prevents high-fat-diet-induced obesity [21, 24], whole body insulin resistance and hyperglycemia in mice by decreasing adipose depots, upregulating adiponectin expression in adipocytes, and modulating inflammatory adipokine expression [21]. However, relatively scarce literature is available on the properties of 20E as a treatment for multiple phenotypic characteristics of CMS. Importantly, there is still a lack of evidence showing whether 20E alleviates CMS features or glucose transport activity in insulin-resistant skeletal muscle. We have recently demonstrated that ovariectomized (OVX) rats fed a high-fat-high-fructose diet (OHFFD) represented a condition of menopausal women with CMS and skeletal muscle insulin resistance [25]. Therefore, this study was designed to investigate the effects of chronic treatment with 20E on insulin-mediated skeletal muscle glucose transport activity and other phenotypic characteristics of CMS in rats with metabolic dysfunction induced by a high-caloric diet after deprivation of female sex hormones.

## Methods

### Animal studies

Female Sprague-Dawley rats at 8 weeks of age, weighing approximately 180–200 g, from the National Laboratory Animal Center, Mahidol University, Salaya, Thailand, were arrived on a strict hygienic conventional housing unit at the Center of Animal Facilities, Faculty of Science, Mahidol University. The temperature was maintained at 22 °C with a 12/12-h light/dark cycle. For concise determination of food and water intake, animals were individually housed in a 9 × 12 × 6 in. hygienic hanging cage with corn cob bedding. Forty-six animals were randomly separated into 6 groups: sham (*n* = 8), OHFFD (*n* = 8), OHFFD treated with 20E (5 mg/kg, 10 mg/kg, 20 mg/kg) (*n* = 7, *n* = 8, *n* = 8), and OHFFD treated with PIO (*n* = 7). The sample size was calculated from insulin-mediated glucose transport data according to Prasannarong et al., 2012 [26] by using Minitab 14 (Minitab Inc., State College, PA, USA). The baseline data on blood pressure, body weight, and food and water intake of each animal were recorded. During the experimental period, the amount of food and water consumed as well as the body weight of each animal were measured. At 10 weeks of age, ovariectomy or sham surgical procedures were performed under anesthesia through bilateral skin incisions at the lower back, as previously described [27]. A mixture of Tiletamine-zolazepam (Zoletil 50; 25 mg/kg body weight) plus xylazine (3–5 mg/kg body weight) was intraperitoneally injected to put the animals into deep anesthesia. Provision of analgesic drug was used for one week after the surgery. Sham rats were fed a control diet (TD08806, 10% kcal as fat; Envigo [formerly Harlan Teklad], Cambridgeshire, UK) with reverse osmosis water. OVX rats were fed a high-fat diet (TD06414, 60% kcal as fat; Envigo) with 30% w/v fructose in drinking water for 12 weeks ad libitum. From the 4th week of the experiment, the treated group was daily given 20E (5 mg/kg, 10 mg/kg, 20 mg/kg) or PIO (10 mg/kg) diluted in 25% propylene glycol by gavage at 0900–1000 h. These concentrations of 20E had been used in previous studies [19, 21, 23]. The animals that received vehicle treatment were orally administered 25% propylene glycol (TCI, Tokyo, Japan). Body weight, food intake and water intake were assessed 3 times a week throughout the 12 weeks of feeding. The total caloric intake of each animal was calculated using the average daily food and water (or fructose solution) consumed and multiplying by the calorie content of the respective diet.

### Blood pressure measurement

A noninvasive computerized tail-cuff system (CODA Monitor System, Kent Scientific Corporation, Torrington, CT) with the indirect tail-cuff method was used to measure systolic blood pressure (SBP), diastolic blood pressure (DBP) and mean arterial pressure (MAP) at baseline and at the 12th week of the experiment in conscious rats. The reported values for each rat were the mean of 10 consecutive readings.

### Determination of whole-body insulin sensitivity

After an 11-week period of treatment, an oral glucose tolerance test (OGTT) was performed. Approximately 15 h before the test, all animals were food restricted (4 g of food), and the fructose solution was replaced with reverse-osmosis water. On the day of the test, ~ 24 h after the last treatment, tail blood was collected before glucose feeding (1 g/kg body weight) by gavage and 15, 30, 60, and 120 min after the glucose challenge. Blood samples were mixed with the anticoagulant EDTA and centrifuged at 13,000 *g* and 4 °C for 1 min. Plasma was kept at 80 °C and used for the determination of glucose (Gesellschaft für Biochemica und Diagnostica, Wiesbaden, Germany) and insulin (Linco Research, MO, USA). Immediately after the OGTT, each animal was given 2.5 ml of sterile 0.9% saline to substitute the lost fluid. The homeostatic model assessment of insulin resistance (HOMA-IR) score was calculated to reflect the whole-body insulin sensitivity from fasting glucose and fasting insulin at baseline. The glucose-insulin (G-I) index was calculated as the product of glucose and insulin areas under the curves (AUCs) and was used to indicate whole-body insulin sensitivity, with a high G-I index indicating low whole-body insulin sensitivity.

### Blood and tissue collection

All animals were food restricted (4 g of food), and the fructose solution was replaced with reverse osmosis water 15 h before euthanasia. Rats were weighed and then anesthetized with an intraperitoneal injection of pentobarbital sodium (nembutal; 75 mg/kg body weight). Soleus muscles were isolated and prepared for in vitro assessment of insulin-mediated muscle glucose transport activity. The plantaris muscle was collected and weighed for representation of lean body mass. Blood was drawn from the abdominal artery, allowed to clot, and centrifuged at 3000 *g* and 4 °C for 15 min to obtain serum samples. The liver and visceral fat pads were excised and immediately weighed. All liver samples were collected from the same lobe of the liver for each animal and were frozen in liquid nitrogen. The uterus was removed and weighed for confirmation of a complete ovariectomy, and the heart was removed to euthanize the animals. Serum and tissue samples were stored at − 80 °C until analysis.

### Assessment of insulin-mediated muscle glucose transport activity

Insulin action on glucose transport activity was determined in insulin-mediated conditions using 2-deoxy-[*3*H]-glucose (2-DG) uptake. The soleus muscle was isolated, divided into 3 portions, and incubated for 60 min at 37 °C in 3 ml of oxygenated Krebs-Henseleit buffer (KHB) supplemented with 8 mM D-glucose, 32 mM D-mannitol and 0.1% radioimmunoassay-grade bovine serum albumin (BSA) (Sigma Chemical, MO, USA). One strip of the soleus muscle was incubated in the absence of insulin, and the other strip was incubated in the presence of 0.2 mU/ml and 2 mU/ml insulin (Human R; Eli Lilly, IN, USA). Flasks were continuously gassed with a mixture of 95% O_2_ and 5% CO_2_ throughout the incubation and transport study procedures. After the first incubation period, each muscle strip was rinsed for 10 min at 37 °C in 3 ml of oxygenated KHB containing 40 mM D-mannitol, 0.1% BSA and insulin (for the muscle strip incubated in the presence of insulin). Each muscle strip was then incubated for 20 min at 37 °C in 2 ml of KHB containing 1 mM 2-[1,2-^3^H]deoxyglucose (2-DG) 300 μCi/mmol; PerkinElmer Life Sciences, Boston, MA), 39 mM [U-^14^C]mannitol (0.8 μCi/mmol, PerkinElmer Life Sciences), 0.1% BSA and insulin (for the muscle strip incubated in the presence of insulin). At the end of the incubation period, muscle strips were removed and trimmed of excess fat and connective tissue, immediately frozen with liquid nitrogen and weighed. Frozen muscles were solubilized in 0.5 ml of 0.5 M NaOH, and 8 ml of scintillation cocktail was added (Ultima Gold; PerkinElmer Life Sciences). Specific intracellular accumulation of 2-DG was determined as previously described [28] using mannitol to correct for extracellular accumulation of 2-DG. Glucose transport activity was measured by determination of the intracellular accumulation of 2-DG (pmol/mg muscle wet weight/20 min). Insulin-mediated glucose transport was calculated as the net increase in 2-DG uptake above the basal level due to insulin.

### Immunoblotting analysis

Frozen rat liver tissue (50 mg) was homogenized using a TissueLyser LT (QIAGEN, Valencia, CA, USA) in 500 μl of ice-cold RIPA lysis buffer (Thermo Scientific Inc., Waltham, MA, USA) containing Halt protease and phosphatase inhibitor cocktail (Thermo Scientific Inc.). Liver homogenates were centrifuged at 12,000 *g* and 4 °C for 20 min, and the protein concentration was determined using the BCA Protein Assay Kit (Pierce, Rockford, IL, USA). Fifty micrograms of protein from each sample was separated using 8–10% SDS-PAGE and blotted onto 0.45 μm nitrocellulose membranes (Bio-Rad, Richmond, CA, USA). Blots were blocked with 5% Omniblok nonfat dry milk (AmericanBio, Inc., MA, USA) in tris-buffered saline (TBS) plus 0.1% Tween-20 for 2 h and incubated with the following primary antibodies at 4 °C overnight: FOXO1 (1:1000, Santa Cruz Biotechnology, TX, USA), pFOXO1 Ser^256^ (1:1000, Santa Cruz Biotechnology), Akt (1:800), pAkt Ser^473^ (1:800), AMPKα (1:800), pAMPKα Thr^172^ (1:800), FGF21 (1:1000, Abcam, Cambridge, UK) and GAPDH (1:3000). Blots were subsequently incubated with horseradish peroxidase (HRP)-conjugated goat anti-rabbit IgG (1:3000) or HRP-conjugated goat-anti mouse IgG secondary antibody (1:3000) at room temperature for 60 min. Antibodies without a supplier indicated were purchased from Cell Signaling Technology (Beverly, MA, USA). Protein bands were visualized by enhanced chemiluminescence (PerkinElmer Life Sciences) using a C-DiGit Blot Scanner (LI-COR Biotechnology, Lincoln, NE, USA) and were quantified with Image Studio software. Band densities were quantified using ImageJ software (NIH). GAPDH was used as an internal control for normalizing protein expression.

### Statistical analysis

The significance of differences among groups was assessed by one-way analysis of variance (ANOVA) with a post hoc Tukey honest significant difference test. A level of *P* < 0.05 was set to indicate statistical significance.

## Results

### Effect of 20E on total caloric intake and body weight

The OHFFD rats consumed more calories than the sham rats (+ 40%, *P* < 0.05), but there was no significant difference among the 20E-treated groups (Table 1). The final body weight and total body weight gain were also higher in the OHFFD group (+ 33%, + 158%, *P* < 0.05, Table 1) than in the sham group. 20E reduced body weight in a dose-dependent manner but was only significant in the 20E 20 mg/kg group (− 16%, *P* < 0.05). This indicated that 20E has a beneficial effect on controlling body weight without affecting eating habits.

**Table 1:** Initial and final body weights; BW gain; total caloric intake; uterus, liver, and abdominal fat weights; plantaris muscle weight; fasting plasma insulin; fasting plasma glucose; and HOMA-IR score in sham rats fed a control diet and OVX rats fed a HFFD (OHFFD) that received 20-hydroxyecdysone (20E) or pioglitazone (PIO)

	SHAM	OHFFD	OHFFD + 20E (5 mg/kg Bw)	OHFFD + 20E (10 mg/kg Bw)	OHFFD + 20E (20 mg/kg Bw)	OHFFD + PIO (10 mg/kg Bw)
Body weight (BW), g						
Initial BW	225.8 ± 2.3	221.6 ± 3.0	212.0 ± 1.3	215.5 ± 3.4	215.3 ± 4.7	208.9 ± 1.2
Final BW	292.9 ± 3.7	390.4 ± 5.6^#^	366.3 ± 11.5^#^	366.3 ± 5.1^#^	361.4 ± 8.5^#^*	370.9 ± 8.4^#^
BW gain, g	61.9 ± 3.1	158.9 ± 5.2^#^	156.9 ± 9.6^#^	152.6 ± 5.6^#^	133.4 ± 4.8^#^*	161.4 ± 6.9^#^
Total caloric intake, kcal	4394 ± 67	6163 ± 118^#^	6044 ± 164^#^	6377 ± 164^#^	6288 ± 199^#^	6160 ± 190^#^
Uterus weight, mg	741 ± 40.2	92 ± 2.4^#^	91 ± 2.4^#^	96 ± 4.4^#^	93 ± 3.5^#^	85 ± 5.2^#^
Liver weight, g	6.7 ± 0.1	8.5 ± 0.2^#^	8.8 ± 0.2^#^	8.8 ± 0.3^#^	9.0 ± 0.3^#^	7.8 ± 0.4^#^
Abdominal fat weight/ body weight, %	5.7 ± 0.2	6.9 ± 0.3^#^	6.3 ± 0.4^#^	5.7 ± 0.3*	5.8 ± 0.1*	5.6 ± 0.3^*^
Plantaris weight/ body weight, %	0.92 ± 0.12	0.89 ± 0.15	0.86 ± 0.03	0.87 ± 0.02	0.86 ± 0.02	0.82 ± 0.02
Fasting plasma glucose, mg/dl	123.3 ± 2.3	128 ± 4.4	127.2 ± 3.5	125.3 ± 2.4	131.8 ± 1.8	118.5 ± 1.7
Fasting plasma insulin, μU/ml	52.7 ± 4.8	61 ± 4.4	61.4 ± 8.2	58.7 ± 7.6	59.4 ± 5.5	30.5 ± 4.3^*^
HOMA IR score	1.6 ± 0.1	1.9 ± 0.1^‡^	1.9 ± 0.3^‡^	1.8 ± 0.2^‡^	1.9 ± 0.2^‡^	0.9 ± 0.1

Values are means ± SEMs for 7–8 animals/group

Data for comparisons between groups were analyzed using one-way ANOVA followed by Tukey’s post hoc analysis. ^#^*P* < 0.05 vs. sham; ^*^P < 0.05 vs. OHFFD, ^‡^*P* < 0.05 vs. OHFFD+PIO (10 mg/kg)

### Effects of 20E on tissue weights

Estrogen plays a critical role in the regulation of the maturational process of female reproductive organs [29]. Uterus weights in the OHFFD rats were 5-fold lower than that of the sham rats, indicating the effectiveness of the ovariectomy. The uterus weights of the OHFFD rats were lower than those of the sham rats and showed no improvement in 20E-treated rats (Table 1), suggesting that 20E treatment had no estrogenic effect on uterus weight. Central obesity was estimated by the ratio of abdominal fat weight to body weight. When compared to the sham rats, the OHFFD rats had a + 21% increase in abdominal fat mass (*P* < 0.05), which was suppressed by 20E treatment in both the 10 mg/kg and 20 mg/kg groups (− 18%, − 16%, *P* < 0.05, Table 1). However, no significant difference in the ratio of plantaris muscle weight to body weight was observed among the groups.

### Effect of 20E on serum lipid profile

Serum lipid profiles are shown in Table 2. The levels of serum triglycerides were significantly increased by + 96% in the OHFFD rats (*P* < 0.05), and PIO reduced the circulating levels of triglycerides. No significant differences were detected in the levels of total serum cholesterol (TC) among the groups. The OHFFD rats exhibited significant increases in LDL cholesterol (+ 46%, *P* < 0.05) and the LDL/TC ratio (+ 58%, *P* < 0.05) when compared to the sham animals. 20E (10 mg/kg and 20 mg/kg) and PIO treatments reduced the levels of serum LDL cholesterol (− 21%, − 26%, − 15%, *P* < 0.05) and the LDL/TC ratio (− 15%, − 15%, − 10%, *P* < 0.05) in the OHFFD rats. There were no significant differences in the levels of HDL cholesterol or the HDL/TC ratio among the groups.

**Table 2:** Triglycerides, total cholesterol (TC), LDL-cholesterol (LDL), HDL-cholesterol (HDL), LDL/TC, and HDL/TC in sham rats fed a control diet and OVX rats fed a HFFD (OHFFD) that received 20-hydroxyecdysone (20E) or pioglitazone (PIO)

	SHAM	OHFFD	OHFFD + 20E (5 mg/kg Bw)	OHFFD + 20E (10 mg/kg Bw)	OHFFD + 20E (20 mg/kg Bw)	OHFFD + PIO (10 mg/kg Bw)
Triglycerides, mg/mL	29.0 ± 1.7	57.0 ± 6.5^#^	41.9 ± 27.7	34.1 ± 10.5	36.4 ± 4.3	26.5 ± 3.3*
Total cholesterol, mg/dL	102.5 ± 2.5	103.0 ± 2.2	90.7 ± 1.7	92.5 ± 2.3	97.0 ± 5.1	130.78 ± 3.4
LDL-cholesterol, mg/dL	12.7 ± 0.3	19.3 ± 0.5^#^	18.4 ± 0.8^#^	15.1 ± 0.4*	14.9 ± 0.5*	16.0 ± 0.7^#^*
HDL-cholesterol, mg/dL	83.0 ± 2.0	85.3 ± 2.2	82.1 ± 2.0	77.9 ± 2.2	77.7 ± 5.1	78.0 ± 1.9
LDL/TC	0.12 ± 0.002	0.19 ± 0.004^#^	0.20 ± 0.010^#^	0.16 ± 0.004^†^	0.16 ± 0.010*^†^	0.17 ± 0.006*^†^
HDL/TC	0.81 ± 0.03	0.83 ± 0.02	0.91 ± 0.02	0.84 ± 0.3	0.80 ± 0.03	0.81 ± 0.03

Values are means ± SEMs for 7–8 animals/group

Data for comparisons between groups were analyzed using one-way ANOVA followed by Tukey’s post hoc analysis. ^#^*P* < 0.05 vs. sham; *P < 0.05 vs. OHFFD; ^†^*P* < 0.05 vs. OHFFD+20E (5 mg/kg)

### Effect of 20E on arterial blood pressure

Table 3 demonstrates systolic blood pressure (SBP), diastolic blood pressure (DBP) and mean arterial pressure (MAP) at baseline and at 12 weeks, i.e., at the end of the interventions. The OHFFD rats exhibited a significant increase in SBP, DBP and MAP (+ 26%, *P* < 0.05) compared with the same parameters in the sham rats. 20E and PIO evidently normalized blood pressure in the OHFFD rats (*P* < 0.05, Table 3).

**Table 3:** Systolic blood pressure, diastolic blood pressure and mean arterial pressure in sham rats fed a control diet and OVX rats fed a HFFD (OHFFD) that received 20-hydroxyecdysone (20E) or pioglitazone (PIO)

	SHAM	OHFFD	OHFFD + 20E (5 mg/kg Bw)	OHFFD + 20E (10 mg/kg Bw)	OHFFD + 20E (20 mg/kg Bw)	OHFFD + PIO (10 mg/kg Bw)
SBP, mmHg						
Baseline	119.7 ± 2.4	125.9 ± 1.8	123.7 ± 1.0	122.9 ± 1.6	126.9 ± 1.3	119.9 ± 1.4
Week 12	119.7 ± 1.3	153.4 ± 3.1^#^	136.6 ± 2.8*	128.7 ± 3.0*	129.1 ± 2.7*	130.8 ± 5.1*
DBP, mmHg						
Baseline	87.8 ± 4.1	92.2 ± 4.1	92.7 ± 1.3	94.5 ± 2.3	96.8 ± 1.7	86.2 ± 1.7
Week 12	86.1 ± 3.8	114.6 ± 2.8^#^	108.5 ± 2.8*	99.7 ± 2.5*	98.7 ± 3.8*	98.2 ± 5.9*
MAP, mmHg						
Baseline	98.5 ± 3.6	100.6 ± 2.6	104.9 ± 1.7	104.3 ± 1.7	106.5 ± 1.5	98.4 ± 1.1
Week 12	96.9 ± 2.8	126.9 ± 2.6^#^	115.3 ± 3.8*	109.1 ± 2.6*	107.1 ± 3*	108.6 ± 3.1*

Values are means ± SEMs for 7–8 animals/group

Data for comparisons between groups were analyzed using one-way ANOVA followed by Tukey’s post hoc analysis. ^#^*P* < 0.05 vs. sham; *P < 0.05 vs. OHFFD

### Effect of 20E on whole body insulin sensitivity

**Fig. 1:**
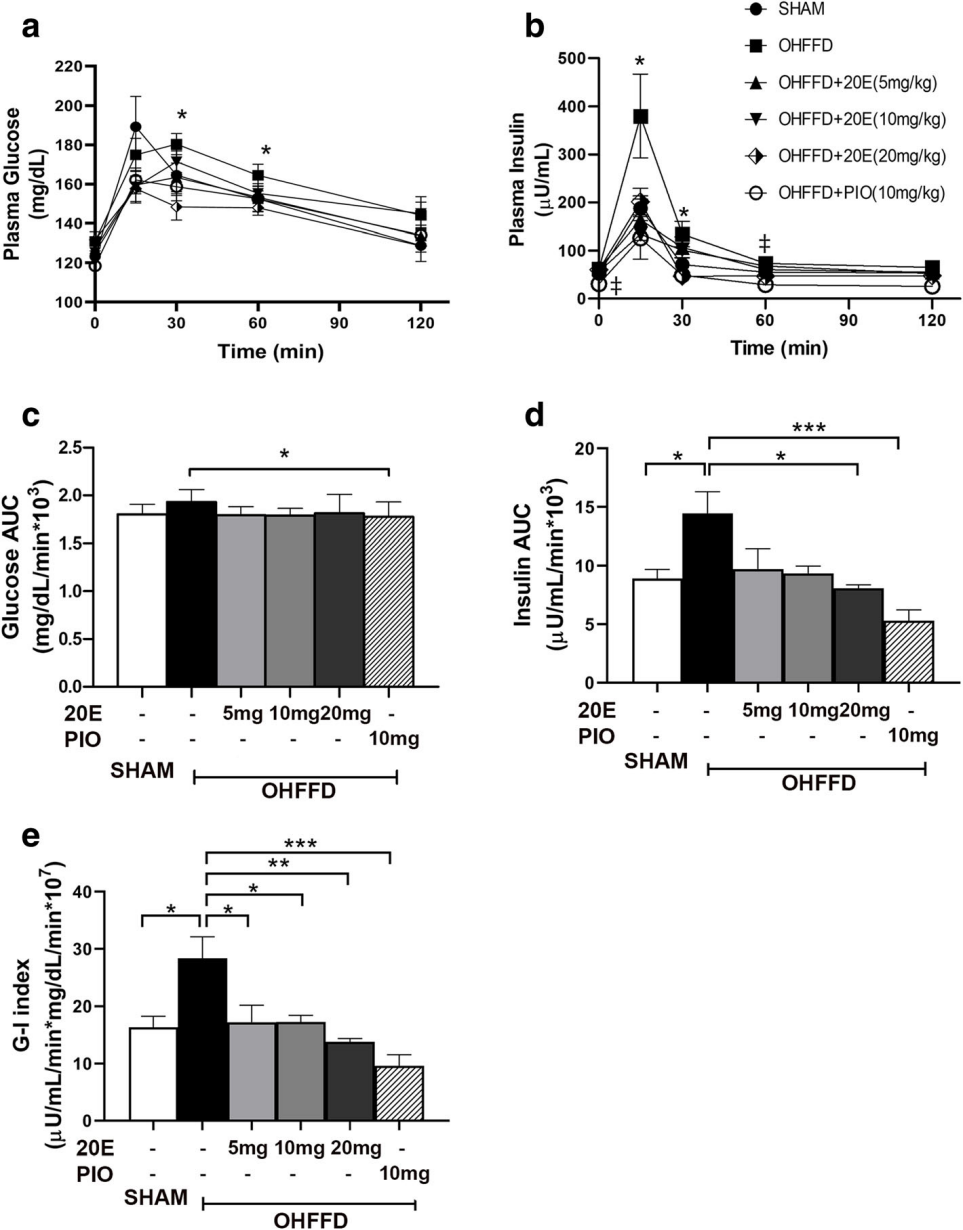
Plasma glucose (**a**) and insulin (**b**) during an oral glucose tolerance test (OGTT), area under the curve (AUC) for glucose (**c**) and insulin (**d**), and glucose-insulin (G-I) index in sham-operated (sham) rats fed a control diet and ovariectomized rats fed a high-fat high-fructose diet (OHFFD) that received 20-hydroxyecdysone (20E) and pioglitazone (PIO). Data for the AUCs were calculated from the glucose and insulin responses. The G-I index was the product of the glucose AUC and insulin AUC for each individual animal. Values are means ±SE for 7–8 animals/group. Data comparisons between groups were performed using one-way ANOVA followed by Tukey’s post hoc analysis. * *P* < 0.05 vs. OHFFD, ‡ *P* < 0.05 PIO vs. OHFFD

The levels of plasma glucose and insulin during fasting are shown in Table 1. There was no significant difference in fasting plasma glucose among the groups. However, fasting plasma insulin showed a significant decrease in the OHFFD+PIO group (10 mg/kg) compared to that in the OHFFD group (− 51%, *P* < 0.05). The 20E-treated groups showed no significant difference in fasting insulin levels. The HOMA-IR score of each rat was calculated based on the relationship between fasting plasma glucose and insulin. The results showed an improvement in insulin sensitivity in PIO-treated rats compared to other OHFFD groups (*P* < 0.05). The levels of plasma glucose and insulin during the OGTTs are shown in Fig. 1. After the glucose challenge, the OHFFD+20E (20 mg/kg) rats exhibited significant decreases in plasma glucose levels at the 30-min and 60-min time points compared to the levels in the OHFFD- group (Fig. 1a), with a 10% decrease in the area under the curve for glucose (glucose AUC) (*P* < 0.05; Fig. 1c) compared with the AUC of the OHFFD rats. Moreover, the insulin levels at the 15-, 30-, and 60-min time points in the OHFFD rats were significantly higher than those of the sham rats (Fig. 1b). Additionally, there was a 66% increase in the insulin AUC in OHFFD group (*P* < 0.05; Fig. 1d) compared to that of the sham rats. 20E treatment at a dose of 20 mg/kg and PIO significantly decreased the insulin AUC by 33% and 60%, respectively, compared with that of the OHFFD rats. The glucose-insulin (G-I) index, the product of the glucose and insulin AUCs, is inversely related to an increase in whole body insulin sensitivity. The G-I index in the OHFFD rats was higher than that in any other group, indicating impaired glucose tolerance and whole-body insulin resistance. The G-I index in the OHFFD rats was effectively attenuated by 20E supplementation and PIO treatment (*P* < 0.05; Fig. 1e).

### Effect of 20E on signaling pathway proteins involved in gluconeogenesis in the liver

**Fig. 2:**
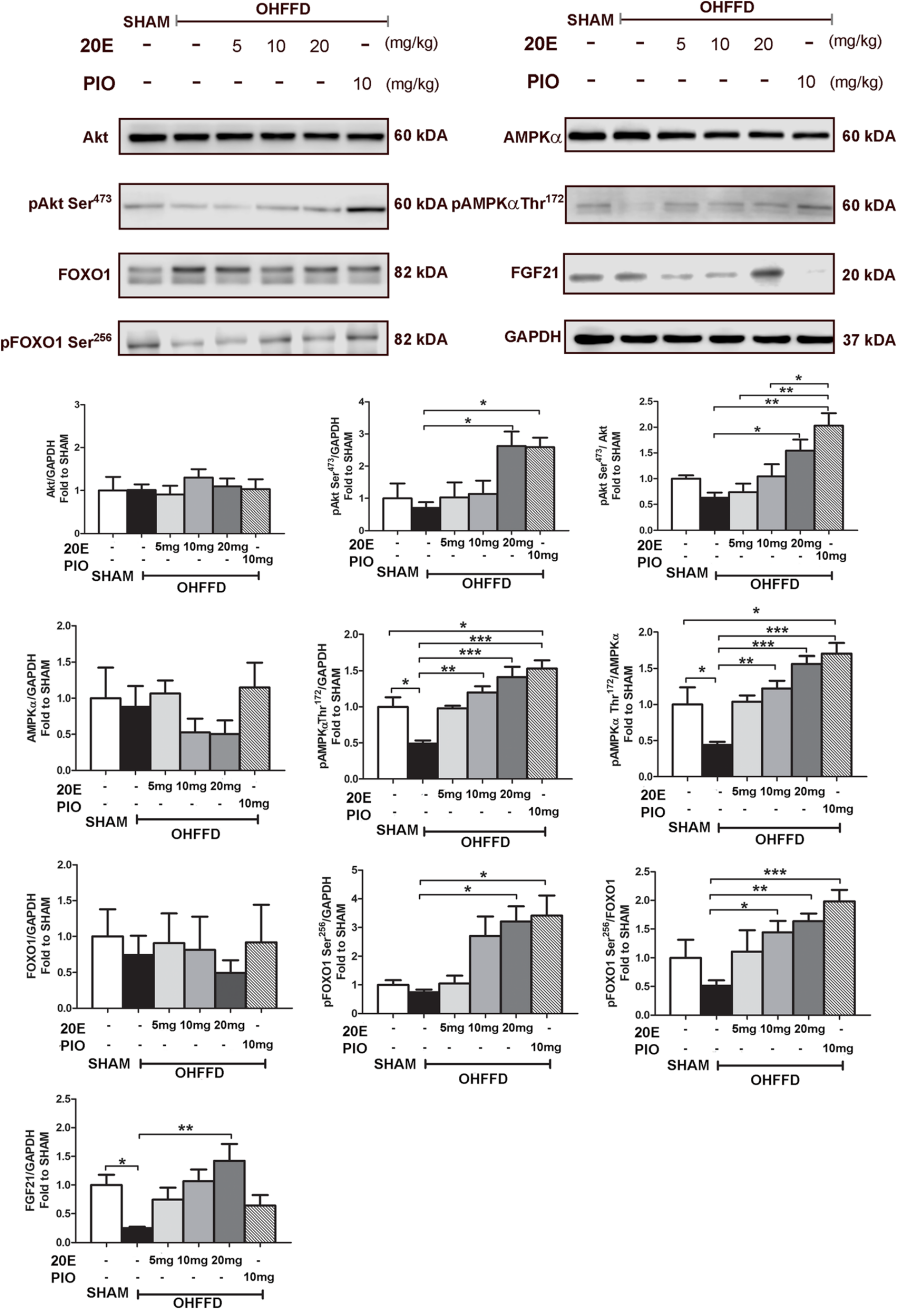
Effects of 20-hydroxyecdysone (20E; 5 mg/kg, 10 mg/kg, 20 mg/kg) and pioglitazone (PIO) on the levels of the following signaling proteins in the liver: Akt, pAkt Ser^473^, FOXO1, pFOXO1 Ser^256^, AMPKα, pAMPKα Thr^172^, and FGF21. The signaling proteins were measured by immunoblotting and normalized to GAPDH. Data are listed as the fold-change in expression levels relative to the sham control group. Representative bands are shown in the top panel. OHFFD: OVX fed a high-fat high-fructose diet. Values are means ± SEM for 7–8 animals/group. Data comparisons between groups were performed using one-way ANOVA followed by Tukey’s post hoc analysis. *P < 0.05, **P < 0.01, ***P < 0.001 between the indicated groups

As FOXO1, Akt, AMPKα, and FGF21 are signaling components contributing to gluconeogenesis in hepatic cells, the activity of these proteins in the liver was evaluated (Fig. 2). The analysis showed no significant difference in the protein expression of Akt, FOXO1, or AMPKα in any groups, as well as no significant difference in the protein expression of pAkt Ser^473^ or pFOXO1 Ser^256^ in OHFFD rats compared to that in sham rats. However, there were significant differences in the protein expression of pAMPKα Thr^172^/AMPKα (− 56%, *P* < 0.05) and FGF21 (− 76%, *P* < 0.05) between the OHFFD and sham groups. Although there was no beneficial effect observed in the 20E (5 mg/kg) treatment group compared to the OHFFD group, the 20E at 10 mg/kg group exhibited increased phosphorylation of FOXO1 Ser^256^/FOXO1 (+ 182%, *P* < 0.05) and AMPKα Thr^172^/AMPKα (+ 147%, *P* < 0.001) and protein expression of FGF21 (+ 184%, *P* < 0.05). The 20E at 20 mg/kg treatment was proven to further increase the activity of pFOXO1 Ser^256^/FOXO1 (+ 267%, *P* < 0.01), pAMPKα Thr^172^/AMPKα (+ 262%, *P* < 0.001), and FGF21 (+ 468%), *P* < 0.01) and showed a significant difference in pAkt Ser^473^/Akt (+ 144%, *P* < 0.05) compared to that in the OHFFD group.

### Effect of 20E on insulin-mediated skeletal muscle glucose transport activity

**Fig. 3:**
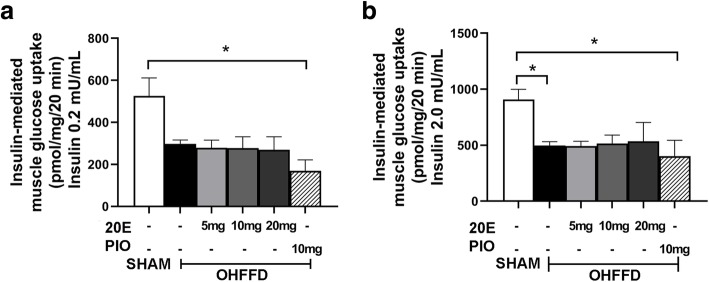
Effects of 20-hydeoxyecdysone (20E; 5 mg/kg, 10 mg/kg, 20 mg/kg) and pioglitazone (PIO) on insulin-mediated glucose transport under 2 insulin concentrations: (**a**) insulin-mediated muscle glucose uptake at 0.2 mU/mL insulin concentration, (**b**) insulin-mediated muscle glucose uptake at 2 mU/mL insulin. Values are means ± SEM for 3–4 animals/group. Data comparisons between groups were performed using one-way ANOVA followed by Tukey’s post hoc analysis. * *P* < 0.05 between the indicated groups

Insulin-mediated glucose transport represents insulin action on glucose transport activity in skeletal muscle, which was calculated as the net increase in glucose uptake above the basal level due to insulin stimulation. There was a significant reduction in insulin-mediated skeletal muscle glucose transport at the 0.2 mU/ml insulin concentrations in the PIO-treated group compared to that in the sham group (*P* < 0.05, Fig. 3a). In addition, there was a decrease in insulin-mediated skeletal muscle glucose transport in the 2.0 mU/ml insulin concentration of OHFFD group (− 40%, *P <* 0.05, Fig. 3b). Nonetheless, no beneficial effects were found in insulin-mediated skeletal muscle glucose transport activity in 20E- or PIO-treated rats. It should be noted that the glucose transport data was excluded if the animals died prior to muscle dissection.

## Discussion

The present study demonstrated that 20E has a favorable effect on whole-body glucose tolerance and alleviates CMS phenotypic characteristics in an HFFD-induced metabolic disorder rat model. This investigation provided novel information that 20E can improve whole body insulin sensitivity in OHFFD rats, and the mechanism may involve the liver. 20E increased hepatic function in glucose homeostasis by activating the protein expression of pAkt Ser^473^, pFOXO1 Ser^256^, pAMPKα Thr^172^, and FGF21. Our study was the first to identify the metabolic effect of 20E on skeletal muscle glucose transport in CMS rats induced by a high-caloric diet after deprivation of female sex hormones. Importantly, this study demonstrated that 20E did not modulate insulin-mediated glucose transport activity in skeletal muscle of OHFFD rats, indicating that the favorable effect of 20E on whole-body insulin sensitivity is not associated with skeletal muscle function. Additionally, we demonstrated that a high dose of 20E treatment reduced CMS characteristics, including abdominal fat mass, LDL cholesterol levels, and blood pressure.

**Fig. 4:**
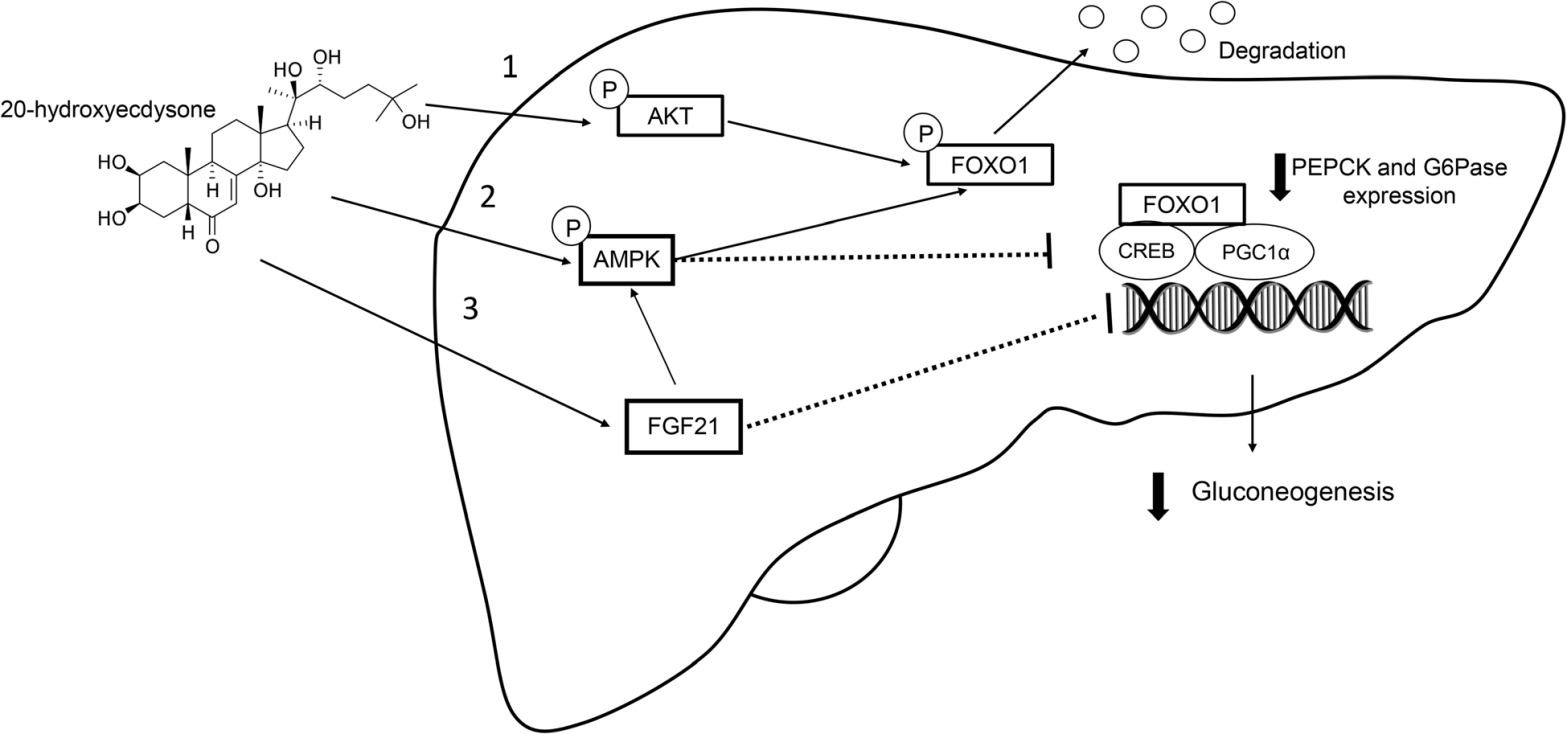
Proposed mechanisms of 20-hydroxyecdysone action in the liver. 20E increases hepatic function by decreasing hepatic gluconeogenesis via both insulin-dependent downstream PI3K/Akt activation and insulin-independent pathways. 20E activates pAkt Ser^473^, which is the IRS substrate in the insulin signaling pathway. The activation of Akt caused the inhibition of FOXO1 to translocate into the nucleus through pFOXO1 Ser^256^. FOXO1 is an important protein that activates PEPCK and G6Pase gene expression. The inhibition of FOXO1 causes a reduction in hepatic gluconeogenesis. 20E also activates AMPKα via pAMPKα Thr^172^, which causes the suppression of CREB action that normally helps stabilize FOXO1 action inside the nucleus. 20E increases FGF21 expression, which then leads to the activation of pAMPKα Thr^172^

In the present study, the OHFFD rat model represented a condition of menopausal women with CMS, including dyslipidemia, hypertension, central obesity, and impaired glucose tolerance along with other characteristics of metabolic abnormalities, such as insulin resistance of skeletal muscle glucose transport activity. The major finding indicated that 20E can improve whole body insulin sensitivity and reduce hyperinsulinemia (Fig. 1). The improvement in whole-body glucose tolerance may be accompanied in part by an increase in the function of insulin-sensitive organs. Several studies in HepG2 [19] and H4IIE [23] cells revealed that 20E can increase hepatic function by increasing hepatic glucose uptake and reduce hepatic glucose production by downregulating PEPCK and G6Pase gene expression through the activation of the insulin-stimulated PI3K/Akt pathway [19, 23, 30]. These two proteins are key enzymes in the hepatic gluconeogenesis pathway. Our findings confirmed that 20E increased hepatic function by decreasing hepatic gluconeogenesis through both an insulin-dependent pathway via the activation of PI3K/Akt downstream and an insulin-independent pathway via AMPKα (Fig. 2). We found that 20E treatment can activate pAkt Ser^473^, which is the IRS substrate in the insulin signaling pathway. The activation of Akt resulted in the inhibition of FOXO1 translocation into the nucleus through pFOXO1 Ser^256^. FOXO1 is an important protein that activates PEPCK and G6Pase gene expression; thus, the inhibition of FOXO1 caused a reduction in hepatic gluconeogenesis. 20E also acted through insulin-independent pathways, such as through its action on AMPKα. 20E activated AMPKα via pAMPKα Thr^172^ (Fig. 2), which in turn suppressed CREB activity and helped stabilize the status and action of FOXO1 in the nucleus. Additionally, as the level of FGF21 expression was increased in the 20E treatment group (Fig. 2), FGF21 may contribute to the suppressive effect of 20E on hepatic glucose production. Even though the function of FGF21 has not been well established, a recent study suggested that FGF21 may regulate energy homeostasis through activation of the AMP-activated protein kinase (AMPK) signaling pathway [31]. These results further indicate that 20E can directly improve hepatic function by suppressing hepatic gluconeogenesis in metabolic disorder rats (Fig. 4). Another important insulin-sensitive organ that helps maintain glucose homeostasis is skeletal muscle. Under feeding conditions, skeletal muscle responds to insulin by increasing muscle glucose transport activity (Fig. 3). However, our study did not find an improvement in muscle glucose transport activity in 20E-treated groups. Arguably due to the action of 20E in skeletal muscle glucose transport, 20E possibly exerted anabolic activity [30] rather than metabolic activity in skeletal muscle.

The improvement of glucose tolerance and hyperinsulinemia observed in this study may lead to further prevention of CMS phenotypic characteristics [32]. Our study showed that 20E helped normalize blood pressure in the OHFFD rats (Table 3). This observation is consistent with the finding that 20E played a protective role against high blood pressure in spontaneously hypertensive rats [31]. Furthermore, we observed that 20E treatment reduced body weight in rats with CMS without affecting caloric intake. Consistent with previous studies [16, 19, 21], the effect of 20E on body weight could be explained by a reduction in abdominal fat accumulation. It should be noted that a reduction in body weight of 20E-treated rats was not associated with any changes in lean body mass, represented by the plantaris weight (Table 1). While the effect of 20E on the serum lipid profile is still controversial [16, 20], our results showed that 20E effectively reduced serum LDL-cholesterol levels without affecting HDL-cholesterol or total cholesterol levels in the OHFFD groups (Table 2).

The molecular mechanisms involved in the 20E-induced effects are not well identified. To date, there are no confirmatory reports about the exact nuclear receptor in mammals that homologs to the 20E nuclear receptor found in insects. Despite other human steroid hormones, 20E did not appear to activate the nuclear androgen receptor (AR), even though the structure is similar to that of androgen steroids [11]. Alternatively, 20E might interact with receptors located on the cell membrane of mammalian cells. Antioxidative effects of 20E on cell membrane lipids have been described [33]. In addition, it has been shown that 20E stimulates the PI3K/AKT signal transduction pathway, which is also activated by insulin-like growth factor 1 (IGF-1). Although calcium-dependent G-protein-coupled mechanism has also been suggested, the associated G-protein has not yet been characterized [34, 35]. The most recent finding proposed that 20E might have an anti-androgenic effect and that it acts through ERβ [36], which was shown in cell culture experiments and induced the hypertrophy of C2C12 cells [33, 34]. ERα is predominantly expressed in reproductive tissues such as the uterus and mammary glands, as well as in the kidney, liver, and heart. Contrary, ERβ is mostly expressed in vascular endothelial cells, the gastrointestinal tract, and the prostate [37]. Nevertheless, both receptors can be coexpressed in a number of tissues and cell types such as the thyroid, epididymis, bone, and regions of the brain. Expression of both ERα and ERβ are demonstrated in the skeletal muscle of mice [38, 39] and humans [40, 41]. Consequently, selective activation of ERβ did not result in any classic estrogen action in this study. 20E did not result in common signs of classic estrogen activity, such as uterotrophy (Table 1). In addition, we found a diabetogenic effect of ERβ activation in skeletal muscle, as there was no improvement in skeletal muscle insulin-mediated glucose transport in 20E-treated rats. Our results support the theory that 20E may act through ERβ in mammals.

Broad-target pharmacological agents that modify insulin resistance are a good initial choice for CMS patients. PIO, a commonly prescribed drug for CMS patients, has been proven to be effective in alleviating metabolic dysfunction [9, 10]. PIO is a synthetic ligand of peroxisome proliferator-activated receptor-γ (PPARγ), which is a member of the nuclear hormone receptor family and contributes to many biological processes, such as glucose regulation and lipid metabolism. We observed that PIO can alleviate metabolic dysfunction by improving whole body insulin sensitivity via the reduction of hepatic glucose production but independent of the action of skeletal muscle glucose transport. The atypical effect of PIO on skeletal muscle glucose transport in this study may be due to the expression of PPARγ being lower in skeletal muscle than in liver and adipose tissue [10]. In this study, PIO prevented the development of other CMS phenotypes, such as abdominal fat accumulation, elevated LDL-cholesterol and triglyceride levels, and high blood pressure. In comparison to 20E, PIO had better efficacy in regulating the whole body lipid profile, especially triglycerides, while 20E had a better ability to activate FGF21 expression in the liver presumably causing suppression of hepatic glucose production in OHFFD rats. Therefore, the extent of insulin resistance reduction and prevention of CMS characteristics in PIO-treated OHFFD rats is somewhat similar to that associated with 20E treatment.

In summary, our findings emphasize the favorable effects of 20E treatment against CMS. 20E mediates improvements in whole-body insulin sensitivity by controlling whole-body glucose homeostasis by modulating hepatic expression, contributing to the proper maintenance of glucose homeostasis, lipid profile, and arterial blood pressure. Therefore, the present study indicates that 20E may be considered an alternative choice for CMS prevention or alleviation of metabolic risk factors beyond lifestyle changes, and further information should be acquired.

## Conclusion

Our findings indicate the beneficial effects of 20E treatment against CMS. 20E treatment proved to be effective in improving whole-body insulin sensitivity by controlling whole-body glucose homeostasis by activating protein expression in the liver (pAkt Ser^473^, pFOXO1 Ser^256^, pAMPKα Thr^172^, and FGF21). In addition, 20E helps control serum LDL cholesterol levels and maintain arterial blood pressure. Therefore, this study demonstrated that 20E may be an alternative for CMS prevention or treatment. It should be noted that an absence of beneficial effects of 20E on insulin-stimulated glucose transport reported in the present study was performed on the soleus muscle (mainly slow twitch fibers). Accordingly, future investigation is required to verify whether the same responses will be observed in other insulin-sensitive tissues, such as adipocytes or muscles with different fiber type composition.

## Data Availability

The datasets used and/or analyzed during the current study are available from the corresponding author on reasonable request.

## Abbreviations

20E: 20-hydroxyecdysone
CMS: Cardiometabolic syndrome
OHFFD: Ovariectomized rats fed a high-fat-high-fructose diet
PIO: Pioglitazone
LDL: low-density lipoprotein-cholesterol
HDL: High-density lipoprotein-cholesterol
TC: Total cholesterol
SBP: Systolic blood pressure
DBP: Diastolic blood pressure
MAP: Mean arterial blood pressure
2-DG: 2-deoxy-[^3^H]-glucose
KHB: Krebs-Henseleit buffer
BSA: Bovine serum albumin
TBS: Tris-buffered saline
HOMA-IR: Homeostatic Model Assessment of Insulin Resistance
G-I index: Glucose-insulin index
AUC: Area under the curve
OGTT: Oral glucose tolerance test
